# Cerebellum as a kernel machine: A novel perspective on expansion recoding in granule cell layer

**DOI:** 10.3389/fncom.2022.1062392

**Published:** 2022-12-21

**Authors:** Hyojin Bae, Sa-Yoon Park, Sang Jeong Kim, Chang-Eop Kim

**Affiliations:** ^1^Department of Physiology, Gachon University College of Korean Medicine, Seongnam, South Korea; ^2^Department of Physiology, Seoul National University College of Medicine, Seoul, South Korea

**Keywords:** cerebellum, expansion recoding, kernel machine, radial basis (RBF) neural network, granule cell layer

## Abstract

Sensorimotor information provided by mossy fibers (MF) is mapped to high-dimensional space by a huge number of granule cells (GrC) in the cerebellar cortex’s input layer. Significant studies have demonstrated the computational advantages and primary contributor of this expansion recoding. Here, we propose a novel perspective on the expansion recoding where each GrC serve as a kernel basis function, thereby the cerebellum can operate like a kernel machine that implicitly use high dimensional (even infinite) feature spaces. We highlight that the generation of kernel basis function is indeed biologically plausible scenario, considering that the key idea of kernel machine is to memorize important input patterns. We present potential regimes for developing kernels under constrained resources and discuss the advantages and disadvantages of each regime using various simulation settings.

## Introduction

Sensorimotor information from multiple sources enters the cerebellar cortex *via* mossy fibers (MFs), where it is transformed and represented in a much larger population of granule cells (GrCs). This “expansion recoding” of MF can be regarded as the projection of distinct patterns into a high-dimensional space where they are more likely to be linearly separable, thus making easier to be readout by Purkinje cells (PC) (Albus, 1971; Cayco-Gajic and Silver, 2019). There have been investigations concerning the computational importance of expansion recoding and what factors contribute it. For example, sparse coding of GrCs has been highlighted as a strategy for producing minimum overlap between GrCs representations for distinct states (Albus, 1971; Marr and Thach, 1991; Olshausen and Field, 2004). Meanwhile, several studies have focused mainly on the role of sparse connectivity between MFs and GrCs in high-dimensional expansion (Billings et al., 2014; Cayco-Gajic et al., 2017; Litwin-Kumar et al., 2017).

However, in majority of these discussions, it has been readily assumed that the input representation is expanded to high-dimensional space *via* random projection. Random expansion can increase the capacity to differentiate across varied input combinations. Meanwhile, it implies that population codes are largely unstructured and task independent (Flesch et al., 2022). Though it is unknown whether the granular layer’s expansion is truly random, a few notable studies have been conducted to examine this subject. According to one study, when noisy clustered inputs were expanded through random synapses, response variability for inputs within the same cluster was increased, and this noise amplification effect was more pronounced as the expanded representation became sparser (Babadi and Sompolinsky, 2014). A recent study, on the other hand, suggested that random expansion in GrC layers can optimally transforms representations to assist learning when compression to pontine nuclei (presynaptic layer to the granular layer) is structured (Muscinelli et al., 2022). There have been also comparable discussions in other brain regions. It has been demonstrated that a high dimensional representation seen in the prefrontal cortex may be produced by non-linearly combining diverse input sources from a randomly connected neuronal population (Barak et al., 2013; Rigotti et al., 2013). Alternatively, it has been shown that the neuronal representations of the prefrontal and parietal cortex are structured on a low-dimensional and task-specific manifold during context-dependent decision making (Mante et al., 2013; Flesch et al., 2022). In the fly olfactory system whose expansion structure is similar with the cerebellum (Stevens, 2015), it is known that glomerular inputs to Kenyon cells increase their dimensions by random convergence rather than organized connectivity (Caron et al., 2013).

In this study, we propose and investigate the possible scenarios of structured expansion of the GrC layer. In particular, we consider the possibility that the GrCs serve as kernel basis functions, thereby the cerebellum can operate like a kernel machine that implicitly uses high dimensional (even infinite) feature spaces. Even highly complex tasks then can be performed by simple linear combination of the granular outputs by the downstream PCs. To investigate candidate scenarios that develop kernels under constrained resources (i.e., restricted number of kernels), we conducted simulations under various experimental conditions and compared their strengths and weaknesses.

## Granule cells can serve as the kernel basis functions tuned to specific combinations of mossy fiber inputs

Although there have been some reports that firing rates of cerebellar neurons linearly encode task-related factors (Ebner et al., 2011), this does not preclude the possibility of the non-linear computations in the cerebellum (Dean and Porrill, 2011). Given the architecture of MFs-GrCs-PCs, GrCs can be modeled as basis functions that mix the MF inputs and offer bases that can be linearly combined by the PCs. Then, mathematically, only simple linear combination in the PC can make non-linear mapping from MF inputs (Raymond and Medina, 2018; Sanger et al., 2020).

**FIGURE 1:**
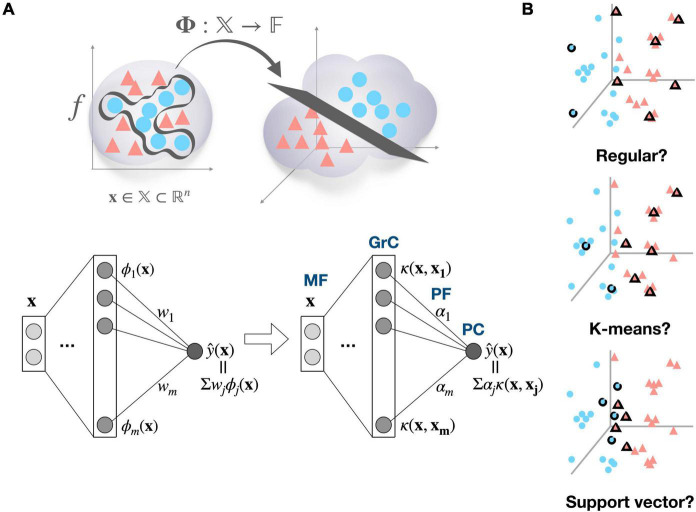
Cerebellum viewed as a kernel machine and candidate regimes for locating kernel basis functions. **(A)** Input data **x** ∈ 𝕏 can become linearly separable by mapping to the high-dimensional feature space 𝔽, and a target function *f* can be expressed as a linear combination of the bases in the high dimensional feature space (top), A learned function of deep neural network $\overset{\wedge }{y}$ can be represented as a linear combination of feature detectors *ϕ*, which compose the mapping function *ϕ*:𝕏→𝔽 (bottom left). If target function *f* exists in reproducing kernel Hilbert space, it can also be found as a linear combination of kernel functions κ:𝕏*x*𝕏→ℝ. Then, the cerebellar microcircuit can be described as a kernel machine, where mossy fiber (MF) inputs are implicitly mapped to the feature space by the kernel evaluations in granule cell (GrC) layer and the learning is confined to synaptic weights between parallel fiber (PF) and Purkinje cell (PC) (bottom right). The *ϕ* subscript and κ subscript denote the index of hidden unit in each network, and *w*_*i*_ and α denote the weight from *j*th hidden unit to the output in each model. **(B)** Representative examples of candidate kernel regime. The regular kernel regime partitions the input space based on Cartesian coordinates. The K-means kernel regime, named after K-means clustering algorithm, covers the data distribution based on their cluster structure. The support vector kernel regime selects samples to maximize the margin between different classes, resulting in a dense allocation of kernel functions along the decision boundary.

Beyond this simple concept of basis function, we propose that a GrC can function as a non-linear kernel (e.g., Gaussian kernel) that computes similarity of the received inputs to a specific activity pattern in the MF space, to which a kernel is tuned. It has been widely observed that neurons in the brain are tuned to important and/or commonly encountered patterns among experienced stimuli (Hubel and Wiesel, 1962; Georgopoulos et al., 1982; Aflalo and Graziano, 2007). Assuming that GrCs are also tuned to previously experienced stimulus patterns, it is natural to view them as kernels memorizing training data samples, which is a fundamental concept of the kernel machine. To elaborate our perspective, first, we gently remind that non-linear decision boundary in the original input space can be transformed to linear decision boundary in high-dimensional feature space (Figure 1A, top). A deep neural network’s learned function can also be expressed as a linear combination of the representation of the last hidden layer, corresponding to the transformed inputs in the feature space (Figure 1A, bottom left). Biological neural networks can also use the same strategy (high-dimensional expansion followed by simple linear combination) to represent complicated functions in many brain areas. However, backpropagation, the standard training method for deep neural networks is often regarded as biologically implausible. The question then becomes how biological neural networks identify high-dimensional feature spaces in order to learn complicated target functions. One possibility is a simple random projection into the high-dimensional space without any structure. The other possibility we propose is that kernel functions can provide efficient solution and the cerebellum could have adopted it. It is well known that every function, no matter how complicated, can be represented as a linear combination of kernel functions as long as it is located in the reproducing kernel Hilbert space (Schölkopf et al., 2002; Figure 1A, bottom right). Kernel functions induce high-dimensional (even infinite) feature space implicitly by memorizing the training samples (known as “kernel trick”). Consequently, given that we assume that kernels already have been properly developed through the evolution or development, the kernel machine can be a biologically plausible model that has the benefit of avoiding computational complexity while utilizing the feature space’s representational capability (Príncipe et al., 2011).

Here, we present a Gaussian kernel as an example of non-linear kernel, which is a popular and powerful kernel for pattern recognition (In the discussion, the biological plausibility of assuming Gaussian kernel is addressed in further depth). If each GrC is thought of as a Gaussian kernel function centered on a certain input pattern, then computation of the granular layer is thought of as a calculation of the similarity between the new input data and the centers of the kernels (i.e., input patterns to which GrCs are tuned). The PCs then compute weighted summation of GrC outputs, and in fact, this circuit is equivalent to a radial basis function (RBF) network. It has been shown that RBF networks with single hidden layer are capable of universal approximation (Park and Sandberg, 1991). However, the problem is that the complexity of kernel methods is often proportional to the number of training data on a linear, quadratic, or even cubic scale. Thus, sparsification, the process of selecting only “important” subset of training samples, is essential. In biological context, limited computing resources might impose this sparsification issue. Consequently, we raise the question of which regime would have been adopted to identify important input patterns (Figure 1B), suggest candidate regimes, and provide preliminary simulation results.

## Candidate regimes for identifying important input patterns under constrained resources

We present potential strategies to identify important input patterns (combination of MFs) and assign them as kernel bases, which could be implemented throughout the evolution and/or developmental process (Figure 1B).

Presented regimes can be broadly divided into a task-independent regime that selects a set of samples that efficiently cover the input space regardless of task demands and a task-dependent approach that selects the relevant samples for the task being performed. Proposed task-independent regimes are as follows; (1) Random kernel regime where samples are chosen randomly, (2) Regular kernel regime where samples are selected in such a way to cover every dimension of the input space evenly, (3) Frequency kernel regime where the most frequent samples are chosen, and (4) K-means kernel regime where samples close to cluster centroids are chosen. Proposed task-dependent regimes are as follows; (1) Support vector kernel regime which pick samples near to the decision surface that separates the classes in a manner that maximizes the margin between the classes, and (2) Novelty kernel regime that sequentially recruits samples with novelty criterion and conducts pruning, alternatively (Refer to “2.5 Method” section for the details). Lastly, random projection regime is adopted as a control.

## Comparing classification performance of candidate regimes under varying conditions

**FIGURE 2:**
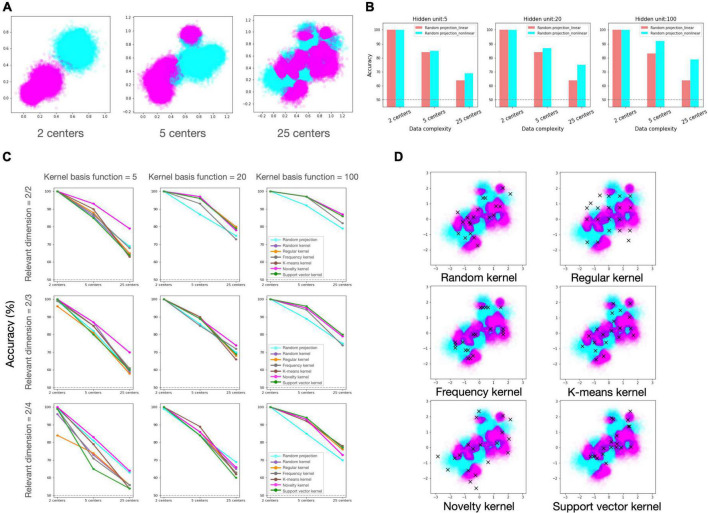
Simulation results of candidate regimes under various experimental settings. **(A)** Generated dataset with varied degrees of distributional complexity. From the left to right: Two classes of data were drawn from a mixture of Gaussian distribution with 2, 5, and 25 clusters. **(B)** Classification accuracy of random projection regime with and without non-linear activation. From the left to right: the performances of network models with 5, 20, and 100 hidden units. **(C)** Classification accuracy of candidate regimes. Each row reflects the proportion of relevant dimensions in input data, and each column represents the number of hidden units (kernel basis functions), reflecting the model’s resource constraint. Each line color represents a different regime, and legends are only inserted in the plots in the last column for visualization. In panels **(B,C)**, the *X*-axis labels depict the number of local clusters in the dataset, representing data distributional complexity. **(D)** Selected samples in each regime. On the input data, chosen samples (centers of Gaussian kernels) in each regime are plotted with the cross mark.

To compare the efficacy of the candidate regimes under diverse experimental conditions, we built neural network models according to each regime and evaluated the learning performances for the binary classification task (Refer to “2.5 Method” section for the details). The experimental condition consists of two components. The first relates to data, which is further subdivided into data distribution complexity (Figure 2A) and proportion of task-relevant dimension. The second is resource constraint, which refers to the allowed number of kernel basis functions that comprise the hidden layer (i.e., the number of recruited GrCs).

First, we confirmed that non-linear computation in expansion layer can provide advantage compared to linear computation in our classification task (Figure 2B). Then, we investigated if the various kernel machines outperform the random projection model under varying conditions, and if so, which kernel regime is preferable under which conditions (Figure 2C). We found that, overall, the kernel regimes outperformed the random projection regime in varying conditions. Among the kernel regimes, the novelty kernel regime was particularly robust to diverse experimental conditions and ranked highly on average. The order of the other kernel regimes was varied depending on the experimental settings, but there was no discernible pattern. The support vector kernel regime rated top in the condition that the largest number of kernels are allowed (kernel basis function = 100), but its performance was suffered greatly under all other conditions. Even if the kernels were chosen in the same way as the support vector machine, one of the most powerful algorithms in machine learning, it may be a highly inefficient strategy when the number of available kernels is insufficient in relation to the complexity of the task. In case of the regular kernel regime, the performance degradation was most noticeable as the fraction of irrelevant feature dimensions grew. This regime distributes kernel resources as uniformly as possible over all dimensions of the input space without regard for data structure or task relevance, therefore when the distribution of data is skewed in a particular direction or relevant information is scarce in the input, this strategy would be extremely inefficient. However, it may be advantageous when the distribution of data is unknown or flexible.

As stated above, kernel regimes generally perform better than random projection, yet it is notable that random projection benefits under certain conditions. The stronger the resource constraint and the sparser the task-relevant dimension (moving to the lower left of Figure 2C), the higher the ranking of random projection. Notably, under the condition that only a few kernel functions are available and that the proportion of task-relevant to irrelevant information in the input is 50/50 (Figure 2C, kernel basis function = 5, and relevant dimension = 2/4), random projection regime came in second, following the first-place method by a small margin. It suggests that random strategy may be effective option in the absence of reliable prior knowledge for the data or task.

## Discussion

The cerebellar GrCs, the most abundant neurons in the brains of many vertebrates, make up the cerebellar input layer. Diverse discussions have been advanced regarding the computational significance of this dense neuronal population. Many studies have focused on expansion recoding and the crucial factors that lead to the high-dimensional representation. These arguments are mostly based on known physiological and anatomical characteristics of the cerebellar circuit, such as coding level of GrCs and sparse connectivity between MFs and GrCs. Our proposal is significant in the following ways: First, we proposed GrCs behave as kernel basis functions, explaining how cerebellar cortex provides great representational capacity at accountable computing cost. Second, this broadens the question to include how to identify the kernel basis functions in terms of resource constraint.

### Related biological features supporting each kernel regimes

Although the kernel regimes we suggested are theoretically driven, there are biological findings to support each regime. First, the regular kernel regime, which disperses neural activity pattern over the coding space, is closely related to the decorrelation between GrC activities serving for the dimensionality expansion of the cerebellar input layer (Albus, 1971; Fujita, 1982; Marr and Thach, 1991). Various studies have been made on circuit-level properties that facilitate decorrelation, including sparse coding (Albus, 1971; Marr and Thach, 1991; Olshausen and Field, 2004), sparse synaptic connectivity (Billings et al., 2014; Cayco-Gajic et al., 2017; Litwin-Kumar et al., 2017), and neuronal non-linearities (thresholding) whose effect is amplified by recurrent connections (Wiechert et al., 2010). There was also a study of cell-level properties that lead to decorrelation between individual neurons; Different types of glutamate receptors on unipolar brush cells, which relay MF inputs to GrCs, contribute to the phase dispersion of GrC response (Zampini et al., 2016). It ensures that a subset of GrC is always active at any point during vestibular input, allowing coding resources to be distributed to cover the entire input space.

The common characteristic of task-independent regimes other than regular kernel regime is to utilize prior knowledge for the input distribution. One significant study suggested that the cerebellum can encode temporal statistics of inputs using Bayesian estimation with a bias toward the mean of the prior distribution (Narain et al., 2018). They demonstrated that principal anatomical and physiological components of the cerebellar circuit for using prior distributions of time intervals are heterogenous temporal basis set across GrCs, learning of synaptic weights onto PCs, and integration of PC activity in the cerebellar deep nuclei. There was also one study whose findings agreed with the concept of frequency kernel regime. They showed that given a population of noisy neurons, each with its own tuning curve, the information-maximizing solution leads to a more precise representation for frequently encountered stimuli (Ganguli and Simoncelli, 2014).

On the other hand, recent experimental findings may lend support to task-dependent regimes in which encoding of task-relevant features takes priority over encoding of task-irrelevant features. According to a one research, GrCs encode more task-related information after learning, and the dimensionality of the neuronal population decreases, indicating an increased correlation (Wagner et al., 2019). This distinct change in neural encoding was observed concurrently in the cortex, implying that shared dynamics emerge and propagate during learning. Though it is largely unknown how feature selection is guided, it is known that during motor learning, the cerebellum can modify ongoing predictive responses based on the widespread predictive feedback signal (Giovannucci et al., 2017), and this feedback may help detect task-relevant inputs. In particular, one recent research suggests that projection from deep cerebellar nuclei to pontine nuclei may be taught in a supervised way by exploiting feedback signal between them to detect task-relevant components sent to GrCs (Muscinelli et al., 2022). In addition, it has been discovered that reward-related signals are transmitted to the cerebellar cortex *via* both mossy and climbing fibers (Kostadinov and Häusser, 2022), and the emergence of GrCs tuned to specific combinations of actions and reward (Wagner et al., 2019) implies that they learn to associate a specific sensorimotor feature with an upcoming reward.

### Compatibility with other models of the cerebellum

Though our experiment is about spatial input pattern classification, it can be broadly interpreted. The input data matrix, in particular, can be viewed as a concatenation of time-varying static inputs, and the GrCs tuned to a particular data sample can then be interpreted as spatiotemporal kernels. Obviously, the data used in our experiments lacks typical temporal structures and it may be needed to examine how the temporal pattern of the input affects our findings in future research. However, we believe the major conclusions drawn from our findings will remain valid.

Given the fact that the GrC layer provides temporal basis filters, the proposed kernel machine can be compared to representative cerebellar microcircuit models such as the adaptive filter model (Fujita, 1982) and the liquid state machine (Yamazaki and Tanaka, 2007). Indeed, both the proposed kernel machine and liquid state machine are analysis-synthesis filters, which belong to a flexible class of adaptive filters in which input signals are decomposed into component signals by a set of filters and then recombined with adjustable weights (Dean et al., 2010). One of the key differences between these models and our kernel machine model is how filters are built. In liquid state machines, the granular layer is modeled as a random recurrent inhibitory network of spiking model neurons (also known as a reservoir), in which temporal integration of recurrent connections for GrC activity makes populations to behave as temporal basis filters with varying time constants. In comparison, we explicitly model the cerebellar circuit as a feedforward network, implicitly assuming that spatiotemporal kernels could be formed as a result of spatial contrast or temporal integration provided by recurrent connections in the granular layer. In other words, feedforward propagation through kernel functions accounts for recurrent connections in the GrC layer. Our proposal to model the cerebellum as a kernel machine is distinctive in that it proposes a specific form of GrC computing, which provides unique insights. Because individual GrCs are modeled as kernel functions tuned to previously experienced stimuli, the discussion is broadened to include which experiences should be retained in the face of limited capacity. Notably, there is a study that models the GrCs as Gaussian kernels across time (Narain et al., 2018), the same as our model. Yet, it was a simplification for the facts that each GrC is likely to be activated at multiple time intervals and exhibit a temporally diverse activity pattern, whereas our focus in introducing the kernel functions was their computational features in terms of kernel machine.

### Biological plausibility for Gaussian tuning of granule cells

The Gaussian kernel we suggested is a popular and powerful kernel for pattern recognition, but the hidden unit in a neural network with a single hidden layer cannot produce Gaussian tuning. Although the GrC layer has typically been modeled as a single layer, we suggest that it be modeled as a multi-layer in order to account for the negative feedback loop between excitatory GrCs and inhibitory Golgi cells. Even though Golgi cell inhibition is included in the cerebellar cortex model, it has typically been treated such that GrCs get homogenous inhibition proportional to the overall activity of the input layer, reflecting the fact that Golgi cells are electrically coupled through a gap junction. Recent observation shown, however, that local Golgi cell circuits exhibit multidimensional population activity during spontaneous behavior, including heterogeneous dynamics localized to a subset of neurons as well as dynamics shared by the whole population (Gurnani and Silver, 2021). This justifies modeling the connections between MFs, Golgi cells, and GrCs on multiple layers. In addition, there were experimental results implying GrC’s Gaussian tuning for specific input patterns. Zebrafish GrCs demonstrated a receptive field spanning 5–25% of the visual field (Knogler et al., 2017), and mouse GrCs exhibited narrow tuning to whisker set point at the level of individual cell while populations seemed to linearly encode a broad range of movement (Chen et al., 2017).

### Two distinct solutions found in artificial neural networks

Proposed candidate regimes for identifying kernel basis functions are divided into task-independent and task-dependent approach, with each approach assuming that the neural representation is organized irrespective of a particular task or according to task needs. Intriguingly, an analogous debate exists in machine learning over the relationship of neural representations with a given task; artificial neural networks address non-linear problems in two distinct ways. One is the so-called lazy regime, in which the hidden layer representation of a neural network is nothing more than a random projection of inputs into high-dimensional space, and only manipulation of readout weight may induce associative learning. Alternately, the network may learn a structured projection to a hidden layer depending on the task requirements (aka rich regime) (Chizat et al., 2019; Woodworth et al., 2020). The random projection model we adopted as a control model corresponds to this lazy regime, and the candidate regimes provided here may be regarded as a compromise between the lazy and rich regimes. The novelty or support vector kernel regime is close to the rich regime since it is supervised to discover task-relevant samples. The task-independent regimes, though they are not random, but they share task-agonistic characteristics with the lazy regimes. The lazy and rich regimes emerge during the training of neural networks when the variance of the weight initialization distribution is high and small, respectively (Chizat et al., 2019). Likewise, the distribution of synaptic weight might affect the degree of decorrelation between GrCs, requiring its incorporation into further experimental validation.

### Biological implications of experimental conditions

Since the sparseness of activation of GrCs is still debated, specifying the number of GrCs recruited for a certain task was challenging in our study. Hence, instead of using the known anatomical ratio of MFs, GrCs, and PCs in constructing the model, we imposed varying levels of resource constraint for the number of hidden units. According to our results, the ideal regime may differ depending on whether GrC coding is sparse or dense.

Typically, input noise referred to the degree to which relevant signals are corrupted by internal or external noise. However, given the diversity of information encoded in the granular layer, including multimodal sensory inputs (Ishikawa et al., 2015), motivation and cerebellar internal state (Badura and De Zeeuw, 2017), the MF input of individual GrCs may comprise both irrelevant and relevant variables to the present task. In this regard, we added an irrelevant dimension to the input in different ratios. Given that evaluation for each regime is often based on the tradeoff between pattern separability and noise robustness, the design of the input noise will be a crucial element.

It has been acknowledged that the number of task parameters will establish the upper limit of the representation’s dimension (Gao and Ganguli, 2015). Though we only adjusted the complexity of data distribution in a single binary classification task, it would be preferable to construct richer tasks and quantify their dimensionality in future research.

### Does decorrelation of hidden units always guarantee high performance?

The decorrelation between hidden units has been recognized as an important determinant of representational dimensionality, which is related to the tradeoff between representational power and generalizability. In our regimes, frequency regime tends to have considerable redundancy between kernel bases. The redundancy can increase precision in a local region where kernel centers are densely distributed. In contrast, regular kernel regime disperses the center position throughout the whole input space, therefore the correlation between kernel bases will be relatively low, hence increasing the capacity for representation (Figure 2D). Despite having the highest dimensionality of the expanded representation, the regular regime was generally graded poorly, and it suffered greatly from a large ratio of irrelevant dimension. This suggests that increasing the dimensionality of a representation by restricting decorrelation does not always ensure optimal performance in all circumstances. The findings of the experiment demonstrated that the task-dependent regimes performed better on average. This result suggests that it could be advantageous to invest redundant resources on precisely encoding the task-relevant information, despite this strategy would not maximize the dimensionality of the representation.

### Kernel machine can be an alternative model of biological neural networks with biologically plausible learning rule

The artificial neural networks were inspired by neural computing and brain structure at first, and the approach that employs them as a computational model of the brain has gained popularity (Richards et al., 2019). However, the backpropagation algorithm’s end-to-end training is a non-biological facet of deep learning, raising the question of how similar the computational concept of an artificial neural network is to that of the brain.

In this regard, backpropagation is not always needed for the kernel basis function to emerge. Indeed, since any target function that reside in reproducing kernel Hilbert space can be represented as a linear combination of kernel functions (Príncipe et al., 2011), learning can be confined to the readout weight alone. Even when utilizing kernel functions centered at randomly picked samples (random kernel regime), the performance in our experiment was quite impressive, indicating the strength of the kernel approach itself. We speculate that Hebbian learning could be a feasible mechanism for the emergence of a kernel function in that the basic idea of reinforcement of a frequently observed pattern is similar. In addition, the learning of the readout layer may be readily taught in a biologically plausible way, such as a covariance learning rule. It is worth noting that expansion coding is seen in diverse brain regions, (Mombaerts et al., 1996; Brecht and Sakmann, 2002; DeWeese et al., 2003; Turner et al., 2008; Chacron et al., 2011) implying that the kernel machine might be a suitable model in a variety of brain regions.

### Closing remarks

We suggested potential regimes and compared their readout accuracy since the purpose of the experiment was to simply demonstrate the pros and cons of them under varying experimental settings. However, representation of each regime might be better evaluated based on broader range of factors (e.g., dimensionality, convergence speed of readout classifier, and computing load). It is also noteworthy that the performance reported here may not be the maximum for each regime. There is room for improvement in performance by doing a more extensive grid search on hyperparameters and using efficient optimization techniques. Also, if adaptive learning was allowed for the width of each Gaussian kernel, the local structure of the data might be captured with more accuracy. The cerebellum, whose characteristic of the circuit is well understood, is a great place to study the computational principle of neural population based on physiological, anatomical, and theoretical reasoning. By actively introducing viewpoints in machine learning, it is not only beneficial to use well-established theories and analytical tools, but also to raise questions from a different angle. Inversely, the solution of biological neural networks may serve as a crucial reference in the field of machine learning.

## Method

### Dataset

We generated datasets in which data from two distinct classes are entangled to varying degrees while distributed in a limited area of a two-dimensional input space. Each dataset was modeled as a mixture of Gaussian distribution with *2K* clusters, parameterized by a mean vector μ_*k*_ ∈ ℝ^2^ corresponding to the cluster centers and an isotropic covariance matrix $\Sigma_{k}=\sigma_{k}^{2}\,{\text{I}}_{2}\in {\mathbb{R}}^{2\,\text{x}\,2}$ corresponding to the cluster covariances, where *K* is the number of clusters in each class (2, 5, and 25 for each dataset) and *k* = 1,…, 2*K*.

For each dataset, 100,000 data points are sampled from the specified distribution, with 70% used as a training set and 30% used as a test set to evaluate performance. The class of each center was randomly assigned, and the class of each sample was determined by the class of the nearest center. Data labels are corrupted independently by random noise with rate 0.05. To mimic the situation that MFs convey diverse information such as external stimuli, interpretation of the context, and internal state of the cerebellum, we added additional input dimensions that have no relevant information to the given task (i.e., irrelevant dimensions). Values for irrelevant dimensions were drawn from the uniform distribution, spanning the same range as values for relevant dimensions.

### Neural network model

The artificial neural model with one hidden layer was constructed. The input is a *p*-dimensional real vector, the hidden representation is a *m*-dimensional real vector, and the output is a scalar (*p* and *m* are varying parameters under experimental conditions). In the random projection regime, the output of the model can be described as follows; $y=\sum_{j=1}^{m}\alpha_{j}\,f\,\left(\sum_{i=1}^{p}w_{i\,j}\,x_{i}+b_{1}\right)+b_{2}$, where α_*j*_ denotes the weight from the hidden unit *j* to the output, *f* denotes the activation function, *w*_*ij*_ denotes the weight from the input unit *i* to the hidden unit *j*, and *b*_*1*_ and *b*_*2*_ denotes the bias of the hidden and output layer, respectively. The elements of projection matrix *w*_*ij*_ were drawn from *N*(0,3^2^), and the readout weights α = (α_1_,…α_*m*_) were determined to be a closed-form solution of the linear least-squares problem in all regimes; α = (***H^T^H***)^−1^***y***, where **H** ∈ ℝ^*n*x*m*^ and **y** ∈ ℝ^*n*^ represent the mean-centered hidden layer matrix and the label vector, where *n* and *m* denote the number of training data and dimension of the hidden layer, respectively. In the kernel regimes, the output of the model can be formulated as follows; $y=\sum_{j=1}^{m}\alpha_{j}\,\kappa \,\left(\cdot ,{\text{x}}_{j}\right)+b,$ where α_*j*_ denotes the weight from the hidden unit *j* to the output, κ(⋅,**x**_*j*_): 𝕏x𝕏→ℝ is Gaussian kernel function centered on **x**_*j*_ ∈ 𝕏; 𝕏 is the input domain, a subset of ℝ^*p*^, $\kappa \,\left(x,{\text{x}}_{j}\right)=\frac{1}{\sqrt{2\,\pi }\,\sigma_{j}}\,e^{-\frac{{\left(x-x_{j}\right)}^{2}}{2\,\sigma_{j}^{2}}}$ for every **x** ∈ 𝕏, where σ_*j*_ is width of the *j*th Gaussian kernel, and they are fixed to 1 for every kernel functions in our experiment. The set of kernel centers $X_{c}={\left\{{\text{x}}_{j}\right\}}_{j=1}^{m}$ was determined by each kernel regime.

### Selecting kernel samples under candidate regimes

The number of allowed kernel functions (the dimension of hidden layer) *m* was varied between 5, 20, and 100, and within this number, *m*-training samples were chosen as the kernel centers for each regime.

The random kernel regime randomly selected *m*-training samples with equal probability. For the regular kernel regime, all dimensions in the input space were binned linearly with a bin size of $\left\lceil \sqrt[{p}]{m}\right\rceil$, where *p* indicates the input dimension. Regular coordinates were then generated by the Cartesian product of discretized dimensions, and 1,000 subsets with *m*-coordinates were sampled. The kernel centers were chosen to be the samples closest to each coordinate. The average of performances for each subset was reported, representing the empirical expected value. For the frequency regime, the input space was divided into a lattice of equal volume subspaces and the number of samples in each subspace was counted (Space division was performed with varying levels of *v*, meaning that the greater this value, the narrower the range for considering similar patterns to be identical). Sorted by the frequency, top *m*-grids were selected and the samples closest to the subspace centroids were chosen as the kernel center. In the K-means kernel regime, K-means clustering algorithm assuming *m*-clusters was applied to the trainset and the closest samples to the captured centroids were chosen as the kernel centers. For the novelty kernel regime, novelty criterion idea (Platt, 1991) was applied with slight modification. The kernel center dictionary began with a single random sample, and training samples are given to the model in a random order. Based on the current set of kernels, the model makes prediction for each incoming sample. If the error has increased by more than the pre-set value δ since the previous stage, it is determined that the novelty (surprise) of that sample is high, and it is added as a kernel center. When the number of centers in the dictionary exceeded *m*, the pruning procedure was used to eliminate the center with the least readout weight whenever a new sample was entered as a kernel center. A grid search across a feasible range was used to choose the hyperparameters (*v* and δ).

## Data availability statement

The original contributions presented in this study are included in the article/supplementary material, further inquiries can be directed to the corresponding authors.

## Author contributions

HB, C-EK, and SJK: conceptualization. HB and S-YP: investigation and writing – original draft of the manuscript. C-EK and SJK: writing – review and editing and funding acquisition. All authors contributed to the article and approved the submitted version.
